# Field cancerization in women without conventional risk factors: insights from a case-cohort study

**DOI:** 10.3389/froh.2025.1653210

**Published:** 2025-08-14

**Authors:** Martina Coppini, Giuseppe Seminara, Rodolfo Mauceri, Olga Di Fede, Gaetano La Mantia, Nicola Mauceri, Valeria Cancila, Vito Rodolico, Giuseppina Campisi

**Affiliations:** ^1^ Department of Precision Medicine in Medical, Surgical and Critical Care, University of Palermo, Palermo, Italy; ^2^ Department of Biomedical and Dental Sciences and Morphofunctional Imaging, University of Messina, Messina, Italy; ^3^ Unit of Oral Medicine and Dentistry for Frail Patients, Department of Rehabilitation, Fragility, and Continuity of Care, Regional Center for Research and Care of MRONJ, University Hospital Palermo, Palermo, Italy; ^4^ Department of Biomedicine, Neuroscience and Advanced Diagnostics (Bi.N.D), University of Palermo, Palermo, Italy; ^5^ Department of Health Promotion, Mother and Child Care, Internal Medicine and Medical Specialties, University of Palermo, Palermo, Italy

**Keywords:** oral cancer, mouth neoplasms, OSCC, field cancerization, risk factors, women, chronic mucosal irritation

## Abstract

**Background:**

Field cancerization (FC) is a well-documented phenomenon in oral squamous cell carcinoma (OSCC), typically reported in patients with known risk habits such as tobacco and alcohol use. To date, limited evidence exists regarding FC in individuals without traditional carcinogenic exposures, as well as in those associated with chronic mechanical trauma. The study aims to report a case series of FC in patients without well-known risk habits observed in the last two years.

**Material and methods:**

This study is a retrospective cohort study conducted at the Unit of Oral Medicine “V. Margiotta” of the University Hospital “Paolo Giaccone” in Palermo (Italy). Between January 2023 and February 2025, a total of 64 patients affected by OSCC were observed. All cases were histologically confirmed through biopsy. For the present study, we focused specifically on the subgroup of patients who developed synchronous and/or metachronous lesions during this period.

**Results:**

A retrospective analysis was conducted on eight female patients (mean age: 75.5 ± 10.3 years) diagnosed with multifocal OSCC. Three patients presented with synchronous lesions, three with metachronous lesions, and two developed both types over time. Six patients (75%) were denture wearers.

**Conclusions:**

This study highlights the relevance of FC in elderly OSCC patients with no history of traditional carcinogenic exposures, except for the high prevalence of denture use, which, however, cannot be considered a clear causal factor. Long-term clinical and radiological surveillance is essential for early detection of multifocal lesions, thereby improving prognosis and patient quality of life.

## 1 Introduction

Oral squamous cell carcinoma (OSCC) is the most prevalent malignant tumor of the head and neck, accounting for approximately 90% of all oral cancers (1–3).

According to GLOBOCAN 2022 data, the global age-standardized incidence rates of oral cancer in high Human Development Index (HDI) countries were 3.8 per 100,000 men and 0.6 per 100,000 women while in low/medium HDI countries they were 10.0 per 100,000 men and 2.2 per 100,000 women. Oral cancer ranks 16th in incidence and 15th in mortality among all cancers, with significant risk factors including ethnicity, lifestyle, and socioeconomic disparities (4).

Traditionally, OSCC has predominantly affected men over the age of 50 with a history of tobacco and alcohol use. Although oral and oropharyngeal cancers are associated with common risk factors (e.g., tobacco smoking, alcohol consumption, betel quid chewing, and Human Papilloma Virus infection), recent trends show a rising incidence among younger individuals, with approximately 15% of cases occurring in patients without well-known risk factors (5–8).

Over the past three decades, developed countries have reported a significant rise in cases among non-smokers, with a special regard among young women. This trend is especially evident in OSCC of the tongue, highlighting the potential role of genetic susceptibility or viral infections, which may contribute to the development of OSCC (9, 10).

Among local contributing factors potentially involved in OSCC pathogenesis, prosthesis-related issues such as ill-fitting or unstable dentures, sharp teeth, and malocclusions warrant particular attention due to their potential role in chronic mucosal irritation (11).

Chronic mucosal irritation may also arise from poor oral hygiene, compromised dentition, or edentulism, and is increasingly recognized as a key factor in promoting a proinflammatory microenvironment within the oral cavity (12).

Although chronic mechanical irritation may be considered implicated in OSCC development, current literature does not establish a definitive causal link. A recent systematic review highlighted low evidence to support an association between chronic mechanical trauma and oral carcinogenesis, emphasizing the need for further well-designed studies to clarify this potential association (13).

The human oral microbiome disbiosis is another potential risk factor for OSCC. Specific oral bacteria, especially periodontal pathogens, may promote carcinogenesis via chronic inflammation, anti-apoptotic effects, and carcinogenic metabolite production (14, 15).

OSCC often arises from oral potentially malignant disorders (OPMDs), a heterogeneous group of oral conditions with variable malignant transformation risk. While not initially cancerous, OPMDs exhibit cellular changes that may progress genetically to carcinoma (16).

Clinically, OPMDs present as white keratotic patches (leukoplakia), red lesions (erythroplakia), or mixed red and white lesions. A key factor in the progression of OPMDs to OSCC is oral epithelial dysplasia (17, 18). Despite this, not all cases of OSCC develop from pre-existing OPMDs (19, 20).

High mortality rates in OSCC are largely due to diagnostic delays, which hinder timely treatment and negatively affect outcomes. These delays often result from both patient and clinician factors, including limited awareness of OSCC clinical features and risk factors. Moreover, the absence of specific signs and symptoms contributes to the diagnostic delay of OSCC, often leading to clinical misdiagnoses (21). Consequently, the 5-year survival rate remains below 50% (22, 23). Also, for this reason, most cases are diagnosed at advanced stages with a high probability of developing secondary tumors. Recurrence, particularly within the first two years, remains a major challenge and significantly contributes to the persistently high mortality rate observed over the past three decades (24–26).

Field cancerization (FC), described by Slaughter et al. in 1953, refers to genetically altered cell clones across multifocal areas that predispose patients to synchronous and metachronous tumors. Adjacent tissue may appear normal but shows cellular atypia, indicating susceptibility to new tumors within the altered field (27, 28).

In OSCC, synchronous tumors arise rapidly at separate sites due to FC, whereas metachronous tumors occur after six months, linked to residual genetic changes in surrounding tissue, reflecting ongoing susceptibility within a genetically altered field (29).

The present observational study aims to report a case-cohort study of OSCC recurrence in FC patients without well-known risk habits and observed in the last two years.

## 2 Materials and methods

The study was approved by the Institutional Local Ethics Committee of the University Hospital “P. Giaccone” of Palermo, Palermo, Italy (approval number #1/2022). The study was conducted during the last two years according to the Principles of the Declaration of Helsinki on experimentation involving human subjects. Written informed consent was obtained from all participants.

The study was performed following the STROBE Statement for Observational Cohort Studies (30).

Patients were attended at the Unit of Oral Medicine “V. Margiotta” of the University Hospital “Paolo Giaccone” in Palermo (Italy) between January 2023 and February 2025. During observation time, a total of 64 patients affected by OSCC were observed. All included patients underwent an oral examination, second-level imaging (e.g., computed tomography, cone beam computed tomography, or magnetic resonance imaging) and an incisional mucosal biopsy to obtain tissue specimens for histological diagnosis.

During the clinical examination, signs of possible mechanical trauma from ill-fitting dentures were identified based on the presence of localized ulcerations, erythematous areas, or pressure marks corresponding to denture-bearing regions.

The clinical diagnostic procedures were performed by expert clinicians: oral biopsy by GC at the Unit of Oral Medicine and histological analysis by VR at the Department of Pathologic Anatomy and Histology of the same University Hospital. Once the diagnosis of OSCC was confirmed, patients were referred to the Units of Oncology and Plastic Surgery for treatment, and they have been periodically monitored at the Unit of Oral Medicine.

For the present study, we focused specifically on the subgroup of patients who developed synchronous and/or metachronous lesions without conventional risk factors for OSCC (i.e., tobacco use and alcohol consumption and HPV infection).

Inclusion criteria were diagnosis of OSCC with synchronous and/or metachronous lesions during the observation time and absence of conventional risk factors (i.e., tobacco use, alcohol consumption, HPV infection); and exclusion criteria included prior or concurrent malignancies in other sites and incomplete clinical or histopathological data.

Oral FC is histologically characterized by the invasive proliferation of atypical squamous epithelial cells penetrating the underlying connective tissue and other features, including nuclear pleomorphism, increased mitotic activity, and variable degrees of differentiation. The diagnosis of FC was based on established criteria, including the presence of distinct oral malignant lesions separated by at least 2 cm of clinically normal mucosa or occurring more than six months apart, in the absence of evidence for local recurrence or metastatic spread. Synchronous tumors were defined as secondary lesions arising at distinct anatomical sites within six months of the primary OSCC diagnosis, while metachronous tumors occurred beyond this interval (29). Both patterns were diagnosed through multiple biopsies and histological confirmation.

Conventional risk factors, including tobacco and alcohol, were recorded by structured interviews and a detailed medical history.

For HPV detection, in addition to the histological analysis of the biopsy sample, p16 IHC examination was also performed and a second section from a fresh sample was sent to the microbiology laboratory for HPV-DNA testing by PCR.

## 3 Results

The main demographic and clinical characteristics of the patients included in the study are summarized in Table 1, while lesion-specific features of OSCC are detailed in Table 2.

**Table 1:** Characteristics of the patients included.

Case	Gender	Age	Patient's habits	Prosthesis	Previous diagnosis of OPMD	Stage	Surgical Treatment	Chemioterapy	Radioterapy	Dead
#1	F	86	No	No	No	IVA (T4aN0M0)	Yes	No	Yes	No
#2	F	72	No	Yes	Yes (PVL)	IVA (T4aN0M0)	Yes	No	Yes	No
#3	F	70	No	Yes	No	IVA (T4aN0M0)	Yes	No	Yes	No
#4	F	78	No	Yes	Yes (PVL)	I (T1N0M0)	Yes	No	No	No
#5	F	86	No	No	No	I (T1N0M0)	No	No	No	Yes
#6	F	62	No	Yes	No	IVA (T4aN0M0)	Yes	No	Yes	No
#7	F	63	No	Yes	Yes (OLP)	I (T1N0M0)	Yes	No	No	No
#8	F	87	No	Yes	Yes (PVL)	II (T2N0M0)	Yes	No	No	No

**Table 2:** Lesion-specific features of OSCC.

Case	Site of 1° OSCC	Site of 2° OSCC	Time between 1° and 2° OSCC (months)	Site of 3° OSCC	Time between 2° and 3° OSCC (months)	Site of 4° OSCC	Time between 3° and 4° OSCC (months)	Synchronous	Metachronous
#1	Right buccal mucosa	Right border of the tongue	1	Left hemipalate	66	Right emipalate	18	No	Yes
#2	Masticatory mucosa in sextant III, masticatory mucosa in sextant IV	Left border of the tongue, left hemipalate	93	–	–	–	–	No	Yes
#3	Right buccal mucosa	Right hemipalate	14	Left buccal mucosa	29	–	–	No	Yes
#4	Left hemipalate, right hemipalate	–	–	–	–	–	–	Yes	No
#5	Masticatory mucosa in sextants IV, V, and VI; masticatory mucosa in sextant II	–	–	–	–	–	–	Yes	No
#6	Left border of the tongue	Masticatory mucosa in sextant V	25	Masticatory mucosa in sextant III	51	Ventral surface of the tongue	5	Yes	Yes
#7	Left buccal mucosa	Masticatory mucosa in sextants III	6	–	–	–	–	Yes	No
#8	Right buccal mucosa	Left buccal mucosa	21	Right lower labial mucosa	0	Right border, ventral surface of the tongue	0	Yes	Yes

A total of eight patients were enrolled in the study, all of whom were female, with a mean age of 75.5 ± 10.3 years (range: 62–87 years). Concerning the OSCC sites, the tongue was the most frequently affected site, followed by the buccal mucosa. Lesions involving the palate were symmetrically distributed between the right and left hemipalate. Additionally, involvement of the masticatory mucosa was observed, with the highest frequency in the lower jaw.

According to the TNM 8th staging classification, OSCC cases were distributed as follows: 3 patients with stage I (3/8; 37.5%), 1 patient with stage II (1/8; 12.5%), and 4 patients with stage IV (4/8; 50.0%).

Regarding life habits of OSCC patients, only one patient (1/8; 12.5%) was a former smoker (not smoked for 20 years), and, worthy of note, signs of possible mechanical trauma due to ill-fitting dentures were observed in six patients (6/8; 75%). All included cases were HPV-negative.

At the time of diagnosis, 4 patients were under follow-up for potentially malignant oral disorders (4/8, 50%), of which 3 were for proliferative verrucous leukoplakia (PVL) (3/8, 37.5%) and 1 for oral lichen planus (1/8, 12.5%).

Seven patients underwent surgical treatment (7/8; 87.5%), while one patient died during the study period (1/8; 12.5%). So, at the time of data collection, seven patients were still under clinical follow-up.

Histopathological analysis of biopsies obtained from mucosal sites both adjacent to and distant from the primary tumors revealed a widespread field of altered mucosa with malignant transformation potential consistent with FC in all cases. The diagnosis of FC was further supported by longitudinal observation of new primary or dysplastic lesions developing over time in different areas of the oral cavity.

**Figure 1:**
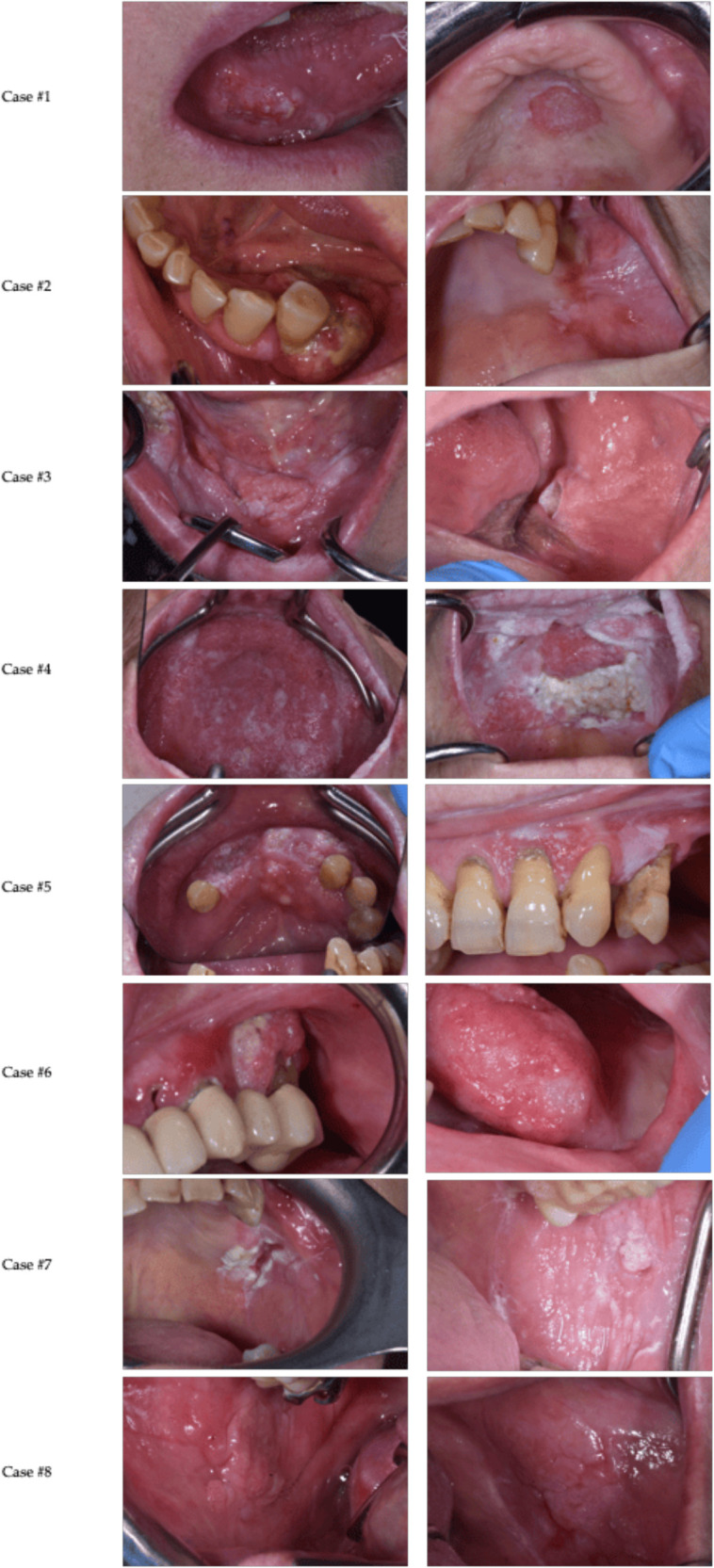
Clinical features of the included cases.

Below, we present a description of 8 cases of OSCC recurrence in FC patients without well-known risk habits, observed from January 2023 to February 2025 (Figure 1).

### Case #1

An 86-year-old woman with a history of multifocal OSCC, previously treated with wide excision of lesions from the right buccal mucosa, right border of the tongue, right floor of the mouth and right labial mucosa, along with right lateral cervical lymphadenectomy and right hemiglossectomy, presented with a new lesion on the palate approximately 4 years later. Intraoral examination revealed an ulcerative lesion with an irregular surface on the left hard palate. Incisional biopsy and magnetic resonance imaging (MRI) of the maxillofacial region confirmed OSCC of the left hard palate. Around 18 months later, the patient developed a new erosive-ulcerative lesion on the right hard palate. Histological examination confirmed OSCC. The patient underwent partial right maxillectomy, extending into the soft palate. Histological analysis revealed OSCC involving muscle tissue and affecting the lateral resection margins, with focal involvement of the medial, posterior, and deep margins. The patient is undergoing clinical and radiological follow-up at another hospital.

### Case #2

A 72-year-old woman, denture wearer, presented with a lesion on the left mandibular region. Intraoral examination revealed an ulcerative mass on the edentulous ridge of the IV-V sextant and left buccal mucosa. Incisional biopsy confirmed the diagnosis of OSCC. The patient underwent hemimandibulectomy, left lateral cervical lymphadenectomy, and postoperative radiotherapy. Histological examination revealed OSCC infiltrating the submucosal connective tissue and left mandibular bone. The patient has been on clinical and radiological follow-up. Nearly 34 months later, the patient presented with proliferative, verrucous white lesions on the masticatory mucosa of the IV-V sextant as well as an atrophic-erosive lesion on the left buccal mucosa. Mapping biopsies were performed, revealing proliferative verrucous leukoplakia on the masticatory mucosa and Cis on the left buccal mucosa. Five years later, an incisional biopsy confirmed the diagnosis of OSCC of the left border of the tongue, left hard palate and recurrence of the left buccal mucosa. She underwent full-thickness excision of the lesions at the Plastic Surgery Unit of our hospital and is currently in close follow-up.

### Case #3

A 70-year-old woman, denture wearer, presented with pain and a non-healing ulcer on the right buccal mucosa. Intraoral examination revealed an ulcerated mass with indurated margins in the same region. Contrast-enhanced MRI of the face and neck demonstrated lesion extension to the right alveolar and masticatory mucosa, along with hyperplastic cervical lymph nodes. Incisional biopsy followed by histopathological analysis confirmed an OSCC infiltrating the full thickness of both mucosal and cutaneous layers, with cortical bone involvement. The patient underwent surgical resection of the lesion, mandibulectomy, and bilateral lateral cervical lymphadenectomy. Fourteen months later, she developed a white verrucous lesion on the right hard palate. Histological examination of a biopsy specimen confirmed Cis, and the lesion was surgically excised. At 29 months post-initial diagnosis, the patient re-presented with a new mass on the left buccal mucosa. Incisional biopsy confirmed recurrent OSCC, and MRI revealed an oval-shaped lesion extending to the fifth sextant. The patient is currently undergoing further diagnostic evaluation and therapeutic management at the Plastic Surgery Unit of our institution and in follow-up at our clinic.

### Case #4

A 78-year-old woman, denture wearer, with a history of proliferative verrucous leukoplakia (PVL) presented with pain and burning sensations on the left border of the tongue. Intraoral examination revealed widespread hyperkeratotic white lesions on the tongue, buccal mucosa, a verrucous mass on the left edentulous ridge and left hard palate, as well as leukoplakic and erythroplakic lesions on the left buccal mucosa and upper lip. Multiple incisional biopsies were performed, and histological analysis showed no malignancy or significant changes. The patient was placed on regular follow-up. Six months later, the patient returned with a new leukoplakic-erythroplakic lesion on the left hard palate, and a biopsy confirmed low-grade dysplasia. Over a year after the last biopsy, a granular, irregularly surfaced mass appeared on both the left and right hard palates. Histological examination confirmed the suspicion of OSCC. The patient was referred for wide excision of the lesion at the Plastic Surgery Unit of our hospital.

### Case #5

An 86-year-old woman presented with lesions on the gingiva of both the upper and lower jaws. Intraoral examination revealed a verrucous exophytic mass on the edentulous ridge of the IV-V-VI sextant and a heterogeneous, hyperkeratotic lesion with erosive areas on the masticatory mucosa of the II sextant. The patient underwent incisional biopsies of both lesions, and histological analysis confirmed the diagnosis of OSCC. Subsequently, the patient underwent facial and cervical MRI with contrast, which revealed an oval mass with irregular margins in the gingival mucosa of the alveolar ridge in the fourth, fifth, and sixth sextants, with invasion into the mandibular bone. The patient was referred to the Oncology and Plastic Surgery Units for further diagnostic and therapeutic management. The patient died six months following the diagnosis of OSCC.

### Case #6

A 62-year-old woman, denture wearer, with a history of left border of the tongue OSCC, presented with new oral lesions. Intraoral examination revealed erosive-ulcerative lesions affecting the left gingival fornix, edentulous ridge in sextants IV-V-VI, and the left buccal mucosa. Histopathological analysis confirmed OSCC of the masticatory vestibular mucosa in sextant V, prompting radical excision. Two years later, leukoplakic and erosive-ulcerative lesions recurred at the same sites, necessitating further excision and mandibulectomy. Histopathological evaluation was negative for OSCC. After another 2 years, a nodular lesion on the left border of the tongue was diagnosed as Cis. Surgical excision confirmed OSCC recurrence, leading to hemiglossectomy and adjuvant radiotherapy. Approximately 9 months post-treatment, the patient developed a granulomatous neoformation on the masticatory vestibular mucosa in sextant III, with histopathology confirming OSCC. Five months later, leukoplakic and erythroplakic lesions emerged on the left vestibular mucosa (sextants V-VI) and ventral tongue. Biopsy and subsequent left maxillectomy with cervical lymphadenectomy confirmed OSCC, involving the retromolar trigone. She remains under close clinical and radiological follow up at our hospital.

### Case #7

A 63-year-old woman, denture wearer, with a prior diagnosis of oral lichen planus (OLP), presented with an ulcerative lesion on the left buccal mucosa and an erythematous area on the left palatal pillar. Histopathological analysis confirmed OLP without evidence of dysplasia. One year later, the patient developed new reticular and erosive-ulcerative lesions on the left buccal mucosa. Biopsy revealed Cis. Surgical excision and reconstruction of the left buccal mucosa were performed, with histological analysis confirming moderately differentiated OSCC. Six months later, a verrucous lesion on the edentulous ridge of the III sextant was biopsied and confirmed as OSCC. The lesion was surgically excised in its entirety. Two months later, during a follow-up visit, the patient presented with a new ulcerative lesion on the left hard palate. MRI showed that the lesion also involved the left maxillary sinus. The patient was subsequently referred to the Otolaryngology department of our institution for complete excision of the lesion, which confirmed the new OSCC. The patient is currently under follow-up at our hospital.

### Case #8

An 87-year-old woman, denture wearer, with a prior diagnosis of PVL with high grade dysplasia of the right buccal mucosa presented with painful new intraoral lesions. Intraoral examination revealed verrucous exophytic lesions on the right buccal mucosa and an ulcerative lesion on the floor of the mouth and right ventral tongue. Multiple biopsies confirmed a diagnosis of OSCC of the right buccal mucosa. The patient underwent wide excision of the lesion with ipsilateral neck dissection. Histopathological analysis confirmed OSCC, moderately differentiated. Two years later, the patient developed other verrucous and ulcerative lesions on the right lower labial mucosa, right lingual margin, and left buccal mucosa. Histopathological examination confirmed the presence of new OSCCs in these locations and the lesions were excised entirely at our hospital. Given the patient's history of PVL, prior OSCC, and extensive surgical treatments, she is under clinical and radiological surveillance every two months to monitor for further recurrences or new oral lesions.

## 4 Discussion

To the best of our knowledge, a limited number of studies have been published on the FC and most of which are case report involving patients with known risk habits such as tobacco and alcohol consumption (2, 31).

The present study is one of the first case-cohort studies to report a case series of field cancerization in patients without exposure to well-known risk habits.

OSCC has a multifactorial origin, with various factors contributing individually or synergistically to its development and is primarily associated with chemical factors (e.g., tobacco and alcohol) and genetic alterations (32, 33).

However, recently, a study investigating the prognosis of non-smoking and non-drinking OSCC patients has been published. In this study, the authors reported that non-smoking and non-drinking OSCC patients possess a 3.9-fold increased risk of developing second primary tumors compared to smoking and/or drinking OSCC patients (34).

Besides well-known risk factors, other variables such as female sex-related susceptibility, especially in advanced age, and prior history of OPMDs may also contribute to the pathogenesis of OSCC (35, 36).

### 4.1 Gender susceptibility

Most studies report a significant gender disparity in OSCC incidence, with a male-to-female ratio of approximately 4:1. Nonetheless, the primary risk factors for OSCC remain consistent across genders, with tobacco use and alcohol consumption being the most significant (37–39).

Despite the limited sample size, our series consisted exclusively of women. This disparity may suggest a potential gender influence in the pathogenesis of OSCC, possibly linked to gender-specific genetic mechanisms.

Females may be more susceptible to FC due to the combined effects of estrogen-mediated epithelial proliferation, differential expression of X-linked genes, sex-specific differences in carcinogen metabolism, and heightened immune and inflammatory responses that may result from female-biased regulation of innate and adaptive immunity, all of which contribute to the persistence and clonal expansion of genetically altered epithelial fields (40).

According to Jayam et al. (2010), individuals presenting with multiple oral lesions may exhibit distinct genetic susceptibility, including reduced DNA repair capacity and increased mutation rates. Gender-related differences, particularly in females, may influence the frequency and severity of these alterations through mechanisms such as hormonal factors, gene expression, immune system variability, epigenetic modifications and environmental exposures (41).

While some studies have shown an increase in OSCC among younger women, our findings highlight a different but equally under-recognized subgroup: elderly women without well-known risk habits, presenting with recurrent or multifocal OSCC (9, 42).

Notably, Karuveettil et al. also reported a higher prevalence of OSCC in women compared to men among non-smokers over the age of 50. This difference may be associated with various risk factors, including chronic mucosal irritation, to which women may be more frequently exposed (43).

Regarding oral FC, recent studies, such as the one by Park et al. (2022), have highlighted that most cases occur in females (35).

Another important finding is that three out of eight patients had a prior history of proliferative verrucous leukoplakia (PVL), a condition known to occur more frequently in female patients. Therefore, the results should be interpreted with caution, as the prevalence of OSCC observed in these women may be partly attributable to this underlying clinical condition (36).

### 4.2 Role of chronic trauma

In the present study, the mean age of the patients was 75.5 ± 10.3 years. This finding aligns with several studies on OSCC, which report an earlier onset in men and a later diagnosis in women, in patients without exposure to tobacco and/or alcohol. Moreover, of the 8 patients included in this study, 6 were denture wearers (75.0%), and only 1 was a former smoker.

Chronic irritation from poor oral hygiene, compromised dentition, missing teeth, and ill-fitting dentures has been proposed as a contributory factor in OSCC development due to its potential to induce persistent mucosal trauma and inflammation (44, 45).

Evidence suggests that chronic trauma may contribute to OSCC development through two primary mechanisms: direct mechanical damage to DNA, supported by increased activity of DNA repair enzymes in affected tissues, and sustained inflammation. The latter involves the release of cytokines and tumor necrosis factor, which drive oxidative stress, leading to genetic and epigenetic alterations. These changes can impair DNA repair, disrupt cellular signaling, inhibit apoptosis, and promote angiogenesis, thereby facilitating carcinogenesis (46–48).

A review by Singhvi et al. demonstrated that chronic mechanical irritation caused by ill-fitting dental prostheses may be considered a risk factor for the development of OSCC, with lesions most commonly occurring on the lateral border of the tongue. However, no significant association was found between the duration of prosthesis use and OSCC incidence (44).

Our data, although in a limited series, seems to support this hypothesis. Most tumors in our series were located on the lateral and ventral surfaces of the tongue, areas particularly susceptible to mechanical irritation potentially caused by factors such as misaligned teeth, poorly fitting dentures, or other sources of chronic mucosal irritation. Additionally, the buccal mucosa was also significantly affected reinforcing the idea that these regions are prone to chronic irritation (46).

### 4.3 Field cancerization mechanisms

Field cancerization theory offers a compelling explanation for the multifocal nature of OSCC. It suggests that carcinogenic exposure or genetic instability affects large areas of the mucosa, leading to multiple primary tumors. Synchronous lesions are diagnosed concurrently or within six months of the primary tumor, while metachronous lesions arise later (29).

The development of OSCC at distinct sites has been explained by three main theories: the theory of independent lesion onset, which suggests that carcinogen exposure induces genetic alterations across multiple areas of the mucosa; the theory of cellular migration, where malignant cells spread either through micro metastasis or intra-epithelial migration; the theory of lesion clonality, which proposes that multiple lesions arise from a single malignant clone (49–51).

In our cohort, three patients presented with synchronous OSCC, three with metachronous lesions, and two developed both types over time.

Some tumors may originate from the same group of damaged cells, while others may develop independently, but all share exposure to carcinogens. FC of the oral cavity suggests that OSCC may not be an isolated event but rather an anaplastic process affecting multiple cells simultaneously. Over time, these abnormal regions may merge, forming larger atypical areas, even after the primary tumor is surgically excised, explaining the emergence of second tumors and recurrences (28).

The FC concept is especially relevant in OSCC, where widespread genetic or epigenetic alterations in the oral mucosa may lead to uncontrolled cellular proliferation and the emergence of tumors at multiple sites. Two models describe this phenomenon: the polyclonal model, in which mutations occur independently at several sites, and the monoclonal model, where mutated cells migrate and establish new lesions from a common origin (31).

Moreover, phylogenetic analysis in previous studies, such as that by Gabusi et al., has demonstrated that histologically normal mucosa can harbor genetically distinct subclones related to the primary tumor, supporting the concept of a genetically altered but phenotypically normal “second field tumor.” This intra-field heterogeneity, also observed among non-neoplastic mucosal samples, suggests that preneoplastic fields are not genetically uniform a finding with potential implications for prognosis and treatment planning (52).

### 4.4 Limitations of the study

The present study possesses some limitations, including the small sample size that restricts the generalizability of our findings. Although all patients in our series lacked conventional risk factors and some had signs of chronic mucosal irritation, our retrospective design does not allow causal conclusions but suggests a potential association worthy of investigation in future prospective studies. Furthermore, the advanced mean age (75.5 years) could also play a confounding role, given the higher prevalence of edentulism and use of dentures in older populations. It is important to consider that approximately half of the included patients had a previous history of OPMD, a factor that may influence the risk of developing OSCC. Moreover, a potential bias of our study is the lack of molecular clonal analysis to determine the monoclonality of multiple neoplastic lesions, limiting our ability to clearly distinguish true second primary tumors, arising independently within a genetically altered field, from local recurrences derived from clonal expansion. Previous studies have successfully used mitochondrial DNA sequencing of the D-loop region to assess clonality and clarify the genetic relationships among lesions (53).

The use of molecular biomarkers offers a promising approach for improving early detection and risk stratification of OSCC, especially in patients without traditional risk factors. MicroRNAs, particularly miR-21, are emerging as non-invasive biomarkers due to their stability in bodily fluids and regulatory roles in carcinogenesis. These markers may aid in identifying genetic alterations in the oral mucosa, enabling targeted surveillance and early intervention (54).

While all patients in our series lacked conventional risk factors and presented chronic mucosal trauma, our retrospective design does not allow causal conclusions but suggests a potential association worthy of further investigation in future prospective studies.

### 4.5 Clinical implications and future perspectives

Despite these limitations, our findings underscore the critical need for long-term surveillance, especially in women without traditional risk habits, as this enables early detection of synchronous and metachronous tumors and contributes to improved clinical outcomes, prognosis, and quality of life.

In this context, as highlighted by Farahani et al., the use of next-generation sequencing and immune profiling may provide valuable insights into the molecular mechanisms underlying secondary primary tumors in non-smoking and non-drinking OSCC patients (55).

Moreover, the implementation of artificial intelligence-based tools may represent a promising approach for the early detection and longitudinal monitoring of OSCC, particularly in patients without conventional risk factors. Recent studies have demonstrated the use of machine learning algorithms for the automated classification of oral lesions, achieving high sensitivity and specificity in distinguishing malignant from benign lesions through clinical imaging data (56, 57).

Finally, chronic mucosal irritation from dental prostheses should receive greater clinical attention as a potential risk factor for OSCC. This perspective may help shift the clinical focus beyond genetic predisposition and toward modifiable environmental factors that remain underappreciated in current practice.

## 5 Conclusions

Field cancerization is a relatively common phenomenon in OSCC patients. The present study emphasizes the need for long-term monitoring in patients with a history of OSCC, given their increased risk of developing new lesions, particularly in the presence of factors including female sex-related susceptibility, advanced age, and the presence of OPMDs.

Despite the absence of traditional OSCC risk factors in this cohort, the predominance of denture wearers among patients with multiple episodes of OSCC raises a potential concern regarding chronic local mucosal irritation as a contributing factor to OSCC onset.

However, due to the limited sample size and lack of molecular data, our findings should be interpreted with caution. Nonetheless, this study offers valuable preliminary information that warrants further investigation in larger cohorts with integrated molecular analyses to better understand the underlying mechanisms and validate these observations.

## Data Availability

The raw data supporting the conclusions of this article will be made available by the authors, without undue reservation.
